# Supplementary material for: A two-hybrid antibody micropattern assay reveals specific in cis interactions of MHC I heavy chains at the cell surface
Source: eLife. 2018 Sep 5;7:e34150. doi: 10.7554/eLife.34150 (PMC6125123; doi:10.7554/eLife.34150)
Supplement: Figure 3—source data 1. [file elife-34150-fig3-data1.docx]

Mean fluorescence intensities for quantification of cluster formation

Table 1: List of mean fluorescence intensities of entire cells and areas of pattern elements used for the quantification of Figure 3B.

| **cell** | **mean fluorescence intensity of entire cell**  **(a. u.)** | **mean fluorescence intensity of patterned areas**  **(a.u.)** | **ratio of fluorescence intensities of**  **patterned area over entire cell** |  | | | **cell** | | | **mean fluorescence intensity of entire cell**  **(a. u.)** | **mean fluorescence intensity of patterned areas**  **(a.u.)** | **ratio of fluorescence intensities of**  **patterned area over entire cell** |  |
| --- | --- | --- | --- | --- | --- | --- | --- | --- | --- | --- | --- | --- | --- |
| **37°C** | | | | |  | | | **+ SIINFEKL** | | | | |  |
| 1 | 99,422 | 118,938 | 1,196 |  | | | 1 | | | 54,131 | 58,620 | 1,083 |  |
| 2 | 60,593 | 89,922 | 1,484 |  | | | 2 | | | 34,644 | 36,610 | 1,057 |  |
| 3 | 97,895 | 133,455 | 1,363 |  | | | 3 | | | 47,883 | 49,074 | 1,025 |  |
| 4 | 71,526 | 99,861 | 1,396 |  | | | 4 | | | 49,407 | 50,923 | 1,031 |  |
| 5 | 46,726 | 63,949 | 1,369 |  | | | 5 | | | 42,123 | 43,988 | 1,044 |  |
| 6 | 44,185 | 62,669 | 1,418 |  | | | 6 | | | 37,450 | 40,890 | 1,092 |  |
| 7 | 63,059 | 83,030 | 1,317 |  | | | 7 | | | 41,460 | 43,607 | 1,052 |  |
| 8 | 64,145 | 70,399 | 1,097 |  | | | 8 | | | 51,859 | 67,480 | 1,301 |  |
| 9 | 30,388 | 37,066 | 1,220 |  | | | 9 | | | 51,370 | 50,919 | 0,991 |  |
| 10 | 38,644 | 52,671 | 1,363 |  | | | 10 | | | 67,442 | 75,861 | 1,125 |  |
| 11 | 99,975 | 115,875 | 1,159 |  | | | 11 | | | 86,878 | 88,246 | 1,016 |  |
| 12 | 45,018 | 65,907 | 1,464 |  | | | 12 | | | 98,137 | 102,829 | 1,048 |  |
| 13 | 20,656 | 27,734 | 1,343 |  | | | 13 | | | 78,565 | 81,861 | 1,042 |  |
| 14 | 127,095 | 141,882 | 1,116 |  | | | 14 | | | 70,749 | 73,212 | 1,035 |  |
| 15 | 35,881 | 43,237 | 1,205 |  | | |  | | |  |  |  |  |
| **Mean: 1,301** | | | | | |  | | | **Mean: 1,067** | | | | |
| **single chain** | | | | | |  | | | **25°C** | | | | |
| 1 | 53,351 | 56,752 | 1,064 |  | | | 1 | | | 39,154 | 43,690 | 1,116 |  |
| 2 | 42,267 | 45,377 | 1,074 |  | | | 2 | | | 109,261 | 125,226 | 1,146 |  |
| 3 | 34,045 | 35,452 | 1,041 |  | | | 3 | | | 89,697 | 89,915 | 1,002 |  |
| 4 | 24,998 | 26,035 | 1,041 |  | | | 4 | | | 150,585 | 154,608 | 1,027 |  |
| 5 | 34,948 | 38,557 | 1,103 |  | | | 5 | | | 66,531 | 71,601 | 1,076 |  |
| 6 | 42,116 | 46,666 | 1,108 |  | | | 6 | | | 81,658 | 82,909 | 1,015 |  |
| 7 | 91,912 | 92,327 | 1,005 |  | | | 7 | | | 90,358 | 98,684 | 1,092 |  |
| 8 | 93,221 | 97,323 | 1,044 |  | | | 8 | | | 41,501 | 44,125 | 1,063 |  |
| 9 | 53,275 | 54,180 | 1,017 |  | | | 9 | | | 79,655 | 90,100 | 1,131 |  |
| 10 | 83,881 | 80,337 | 0,958 |  | | | 10 | | | 68,581 | 73,660 | 1,074 |  |
| 11 | 67,206 | 68,932 | 1,026 |  | | | 11 | | | 80,284 | 88,657 | 1,104 |  |
| 12 | 98,710 | 99,087 | 1,004 |  | | | 12 | | | 74,428 | 75,354 | 1,012 |  |
| 13 | 84,610 | 82,455 | 0,975 |  | | | 13 | | | 66,565 | 75,758 | 1,138 |  |
| 14 | 79,938 | 80,681 | 1,009 |  | | | 14 | | | 76,308 | 86,119 | 1,129 |  |
| **Mean: 1,033** | | | | | |  | | | **Mean: 1,080** | | | | |
